# Sensitive determination of malondialdehyde in rat prostate by high performance liquid chromatography with fluorescence detection

**DOI:** 10.1038/s41598-020-61074-3

**Published:** 2020-03-04

**Authors:** Xiuli Dong, Jiayuan Tang, Xiangming Chen

**Affiliations:** School of Pharmacy, Binzhou Medical University, Yantai, 264003 China

**Keywords:** Lipid peroxides, Assay systems

## Abstract

An excellent pre-column fluorescent derivatization reagent N-acetylhydrazine acridone for the quantitative determination of malondialdehyde was synthesized. Malondialdehyde was derivatized at 80 °C for 30 min in the presence of trichloroacetic acid. The separation of the derivative was performed on an Agilent ZORBAX SB-C18 column in conjunction with gradient elution. The excitation and emission wavelengths were 370 nm and 420 nm, respectively. The developed method demonstrated good linear relationship in the range of 0.02 pmol to 2.5 pmol (r = 0.9998). The calculated limit of detection and limit of quantification were 2.5 fmol and 8.3 fmol, respectively. The analytical precisions of the method were in the range of 1.36–2.27% (intra-day) and 2.36–3.92% (inter-day) respectively. The method was sensitive, specific and simple. It was successfully implemented to analysis the malondialdehyde in rat prostate.

## Introduction

Reactive oxygen species (ROS) can cause oxidative stress that is related to cell aging and plays an essential role in the pathogenesis of a variety of illnesses. During oxidative stress, the double bonds of polyunsaturated fatty acids are peroxided by ROS to yield lipid hydroperoxides, which are readily decomposed into some secondary products by several sequential reactions. One of the major secondary products is malondialdehyde (MDA)^1,2^. MDA is reactive toward proteins and nucleic acids. It has been inferred to have mutagenic and cytotoxic effects by generating DNA-protein cross-links. MDA was found elevated in various diseases including cancers^3^, diabetes^4^, cardiovascular diseases^5^ and liver diseases^6^, making it an indirect indicator of these diseases. Therefore, it is of great significance to determine its content accurately. However, the accurate determination is extremely difficult because of the lower concentration and many interfering factors existing in biological samples^1^. Therefore, a sensitive and selective method for the determination of MDA is needed.

Many methods have been developed for the determination of MDA, mainly including spectrometry^7,8^, gas chromatography-mass spectrometry (GC-MS)^9^, high performance liquid chromatography (HPLC)^10^ and liquid chromatography with mass spectrometry (LC-MS)^11,12^. MDA shows weak absorption in the UV-visible region, so it is very difficult for direct determination of biological sample by spectrometric techniques. To overcome this difficulty, derivatization has been adopted by most methods developed in recent years. The most commonly used derivatization reagent is 2-thiobarbituric acid (TBA) that can react with MDA at 100 °C with acid as catalyst, and the reaction product has strong ultraviolet-visible absorption at 533 nm^13^. However, TBA can also react with many other carbonyl compounds existing in biological samples, which would result in considerable overestimation for MDA^14^. Furthermore, the sensitivity of this method is not high enough for accurate determination of trace MDA in biological samples^15^. HPLC with strong separation capability could improve the selectivity^16^. Due to the weak ultraviolet absorption of MDA, it should be labeled by derivatization reagent. The derivatization reagent used for HPLC-UV is usually 2,4-dinitrophenylhydrazine (DNPH) that can react with MDA at low pH to produce MDA-DNPH derivative^17,18^. The benefit of these methods is that the reaction of DNPH with MDA was conducted at a mild temperature of room temperature^17^ or 37 °C^18^, but the process is slower than the TBA method, and the sensitivity is not high enough to determine the lower concentration MDA in some biological samples. MDA can also be derivatized by diaminonaphthalene (DAN) in an acidic medium at 37 °C^19,20^, and the reaction affords a product (MDA-DAN) with a high UV response at 311 nm. However, the derivatization reaction requires a long derivatization time of 180 min and low pH of 0.8 and the sensitivity of the method is still not high enough for biological samples with MDA at trace level. Because HPLC with fluorescence detection has higher sensitivity than UV detection, which was widely adopted to overcome the disadvantage of low sensitivity. MDA without fluorescence must be labeled by fluorescent reagents. These reagents include TBA and FMOC-hydrazone^15^. FMOC-hydrazone can react with MDA in an acidic medium at 50 °C, but derivatization would consume 4 h to get highest yield, and the mixture should be neutralized with 5.0 mol/L NaOH before injected. The procedure is prolixity and tedious. TBA usually used in the spectrophotometric determination of MDA also has been used in HPLC method with fluorescence detection^21–23^. Some reports have compared the two methods for the determination of MDA^13,24^. The HPLC method with great separation ability can eliminate the interference of compounds present in biological samples, so the selectivity of the HPLC method was better than traditional spectrophotometric method. However, various shortcomings limited the application of these HPLC methods. As a result, a new analysis method for MDA determination is still needed to improve the selectivity, sensitivity and analysis process.

In this study, an excellent fluorescent derivatization reagent N-acetylhydrazine acridone (AHAD) was synthesized. MDA was derivatized by AHAD in acidic medium. The derivation conditions including acid kind, acid concentration, temperature, time and AHAD concentration were optimized to ensure complete reaction of MDA with AHAD. A more sensitive and efficient HPLC method for the determination of MDA based on the derivatization was developed. The proposed method has been applied to determine MDA in rat prostate.

## Experimental

### Chemicals and reagents

**Figure 1:**
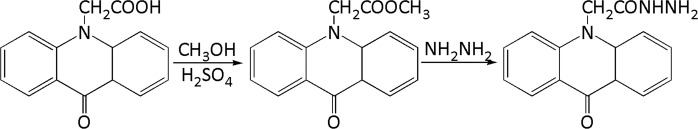
Synthesis route of AHAD.

1,1,3,3-Tetraethoxypropane was obtained from Shanghai Aladdin Bio-Chem Technology Co. Ltd. (Shanghai, China) with a purity of 97%. N-acetylhydrazine acridone (AHAD) was synthesized in the authors’ laboratory (Fig. 1). Acetonitrile and methanol were of HPLC grade and purchased from Anaqua Chemical Supply (Wilmington, USA). Phosphoric acid, hydrochloric acid, sodium hydroxide, dimethylformamide, potassium carbonate, acetic acid, sulfuric acid and trichloroacetic acid were all of analytical grade and purchased from Sinopharm Chemical Reagent Co. Ltd. (Shanghai, China). Acridone was purchased from Maya-reagent (Jiaxing, Zhejiang). Pure water was prepared by Milli-Q super pure water system.

### Equipment and chromatographic conditions

The separation and analysis of MDA were conducted on an Agilent 1260 series HPLC system (Agilent Technologies, USA) equipped with a G1311B quaternary pump, a G1329B autosampler, a G1316A thermostated column compartment, a G1321B fluorescence detector (FLD) and an Agilent ZORBAX SB-C18 column (250 mm × 4.6 mm, 5 μm).

Eluent A and B were water and acetonitrile, respectively. The separation of MDA was accomplished with gradient elution: 0–10 min, 20% B to 45% B; 10–15 min, 45% B to 100% B. The flow rate was constant at 1.0 mL/min. The fluorescent detection wavelengths were set at 370 nm for excitation and 420 nm for emission. The column temperature was kept at 30 °C.

### Synthesis of AHAD

AHAD was synthesized according to the previous method^25^ with a minor revision. 2.53 g of 2-(9-acridone)-acetic acid (10 mmol) and 100 mL of methanol were added to a 250 mL three-neck flask, then, 5 mL concentrated sulfuric acid was added. The mixture was stirred at reflux for 4 h. After the reaction was ended, the solution was poured into ice water. The precipitation was filtered and dried in a vacuum desiccator to obtained 2.38 g of yellow solid (89.1% yield).

1.4 g of 2-(9-acridone)-ethyl acetate (5 mmol) was added to a 100 mL flask, then 30 mL of ethyl alcohol and 10 mL of hydrazine hydrate (85%) were added. The mixture was stirred at reflux for 6 h. After cooled to room temperature, the mixture was poured into ice water with shaking for 5 min. The precipitation was filtered and dried in a vacuum desiccator. The crude product was recrystallized from methanol and dimethylformamide (*v*/*v* 1:3) to obtain yellow crystal (0.91 g, 68.2% yield).

### Preparation of solutions

A 0.01 mol/L of AHAD stock solution was prepared by dissolving 26.8 mg the chemical with acetonitrile up to 10 mL. The low concentration solution (0.5 mmol/L) was prepared by diluting the stock solution with acetonitrile. MDA was obtained by the hydrolysis of 1,1,3,3-tetraethoxypropane in dilute acid solution. 292 μL of 1,1,3,3-tetraethoxypropane was dissolved in 100 mL of 1% sulfuric acid solution to get standard MDA stock solution (0.012 mol/L). The standard MDA solutions for derivatization and HPLC analysis were prepared by diluting the stock solution with water. All solutions were stored at 4 °C until analysis.

### Samples

Experiments were performed on six healthy male rats. The study was reviewed and approved by Ethics Committee of Animal Care and Experimentation of Binzhou Medical University and was performed in accordance with the National Institutes of Health (NIH) Guide for the Care and Use of Laboratory Animals. All rats were decapitated, abdominal dissected, and the prostate gland of each rat was carefully separated and extracted (removal of incidental prostate connective tissue). Prostate gland homogenate was prepared by mixing one portion of 100 mg of prostate gland with 0.9 mL normal saline and homogenized in a mechanically driven Teflon glass homogenizer. The homogenate was centrifuged at 10000 rpm in an automatic high-speed cold centrifuge for 10 min at 4 °C. The supernatant was collected as prostate gland extract and kept in −80 °C refrigerator until analyzed.

### Derivatization procedure

To a 2 mL ampoule, 250 μL of AHAD solution, 100 μL of standard MDA solution and 100 μL of trichloroacetic acid (1.0 mol/L) were added in succession. After sealed, the ampoule was bathed in 80 °C water for 30 min. After the reaction was completed, the mixture was cooled to room temperature. An appropriate volume of water was added to dilute the solution to 1.0 mL, and 10 μL of the final solution was injected into the HPLC system for analysis.

MDA in a sample was derivatized by AHAD after acidic hydrolysis in a one-step reaction. 100 μL of prostate gland extract, 100 μL of trichloroacetic acid and 250 μL of AHAD were mixed. The ampoule was incubated for 30 min at 80 °C. After cooled to room temperature, the solution was diluted to 1.0 mL by water. Then, the solution was transferred to a 2.0 mL Eppendorf tube, and centrifuged at 10000 rpm for 5 min, and 10 μL of supernatant was injected into the HPLC system for analysis.

## Results and Discussion

### Optimization of derivatization conditions

**Figure 2:**
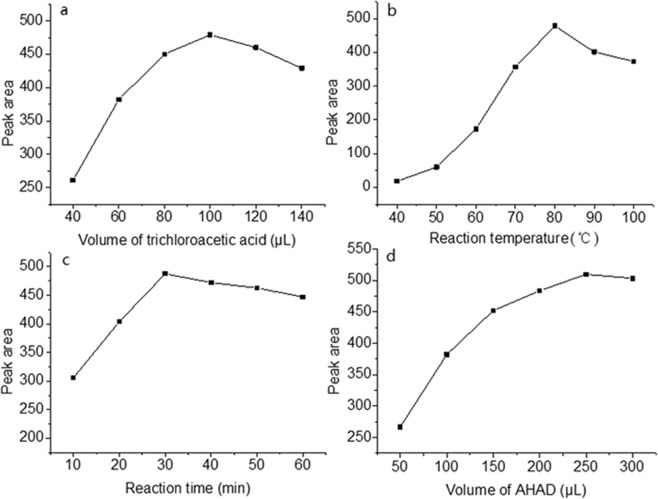
Effect of catalyst (**a**), temperature (**b**), time (**c**) and amount of AHAD (**d**) on derivatization reaction.

To get the highest derivatization response, different acids include acetic acid, formic acid, oxalic acid, citric acid, phosphoric acid and trichloroacetic acid were tested to evaluate the effect of a catalyst on the derivatization. The study of these catalysts is based on previous reports for the determination of fatty aldehydes labeled by 1,2-benzo-3,4-dihydrocarbazole-9-ethoxy-carbonylhydrazine^26^. The maximum peak area was achieved when trichloroacetic acid (1.0 mol/L) was used. So trichloroacetic acid was selected for subsequent experiments. Subsequently, the amount of trichloroacetic acid was investigated over the range of 20–200 μL. The maximum peak area was found when 100 μL of trichloroacetic acid was added (Fig. 2a).

The effect of reaction temperature was investigated in the range of 40–100 °C. The results showed that the peak area of MDA increased with the rise of the temperature, and when it was 80 °C, the peak area was the largest (Fig. 2b). There was an obvious change for the peak area over 80 °C, which may be due to the decomposition of derivative at high temperature. As a result, 80 °C was selected as the derivatization temperature. The effect of the reaction time on the derivative yield was also examined. Based on the results (Fig. 2c), 30 min was selected for the derivatization because the peak area is the largest.

The effect of AHAD concentration was also investigated to ensure MDA completely reacted with derivatization reagent. Different volume of AHAD solutions was tested. The results (Fig. 2d) showed that the peak area was the largest when 250 μL of AHAD was added, and excessive AHAD had no significant effect on peak area. Furthermore, more AHAD would not generate more by-products and interfere with the separation of MDA derivative. Therefore, 250 μL of AHAD was selected, and the derivatization reaction was completed at 80 °C in 30 min.

### HPLC separation

**Figure 3:**
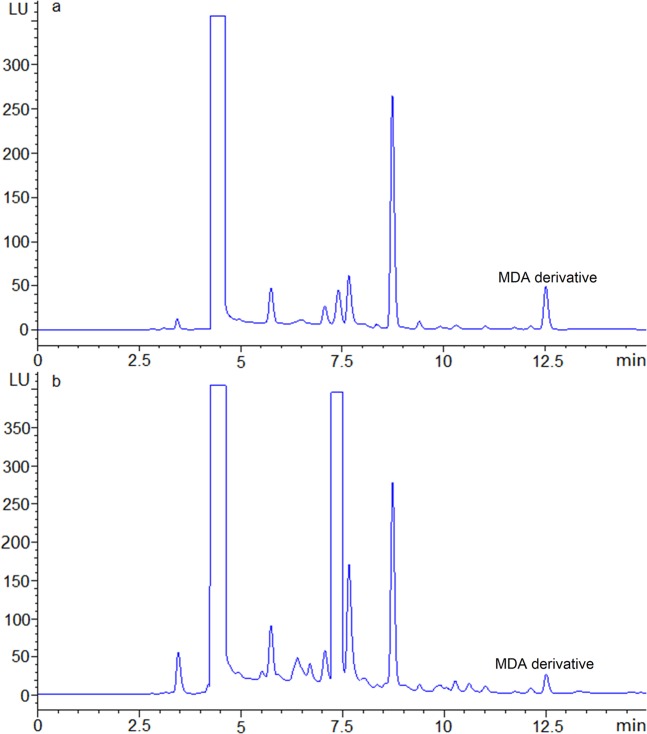
Chromatogram of standard MDA (**a**) and MDA in rat prostate sample (**b**).

To obtain better chromatographic separation with a shorten retention time for MDA derivative, several different chromatographic columns were tested and evaluated for the optimal separation. These columns included Eclipse XDB C18 (250 mm × 4.6 mm, 5 μm), ZORBAX SB-C18 (250 mm × 4.6 mm, 5 μm), and Eclipse Plus C18 (250 mm × 4.6 mm, 5 μm). The results indicated that the ZORBAX SB-C18 column gave the best resolution (R = 1.9) for both standard solutions and sample solutions with the column temperature at 30 °C, however, the resolutions for other two columns were 1.4 and 1.3 respectively. The chromatograms of three columns were shown in Supplement Fig. 1. Therefore, the ZORBAX SB-C18 column was selected for all sequent experiments. Water and acetonitrile were used as eluent A and B respectively, to provide an efficient separation in conjunction with the gradient elution mentioned in the experimental section. The retention time of MDA derivative was 12.52 min (Fig. 3a).

### Stability of AHAD and MDA derivative

The stability of AHAD has been studied in previous work^25^. It was stable enough when stored at 4 °C, and no obvious change for the derivatization efficiency was observed in a week. The stability of MDA derivative was determined by reanalysis of the same derivative solution. The solution was stored at a 4 °C refrigerator when not analyzed. The percent change of peak area was less than 2.7% in 48 h, which indicated the derivative of MDA with AHAD is very stable over 2 days.

### Selectivity of the method

To evaluate the selectivity of the developed method, some aldehydes possibly found in rat prostate was investigated. These aldehydes included formaldehyde, glyoxal, acrolein and heptanal. A standard MDA solution spiked with these aldehydes (100 nmol/mL) was prepared and analyzed according to the procedure described above. The peak area of MDA had no obvious change compared with the standard MDA solution not spiked these aldehydes, which mean the existence of other aldehydes would not disturb the separation and quantization of MDA in rat prostate. The developed method showed good selectivity.

### Linearity, correlation coefficients, detection limits and quantification limits

**Figure 4:**
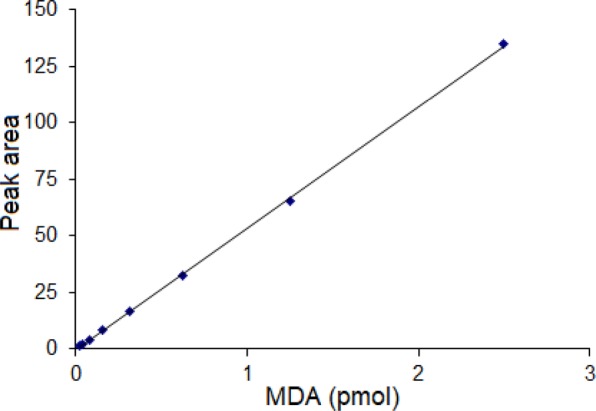
The calibration curve of MDA.

The linearity was established by the analysis of MDA standards derivatized with AHAD following the procedure described above. MDA derivative gave an excellent linear response over the range of 0.02 to 2.5 pmol with correlation coefficient of 0.9998. The calibration curve of MDA was shown in Fig. 4. The linear regression equation for MDA was Y = 53.71 × −0.30 (X: injected amount/pmol, Y: peak area). The calculated limit of detection (LOD) (S/N = 3) was 2.5 fmol (0.25 nmol/L), and limit of quantification (LOQ) (S/N = 10) was 8.3 fmol (0.83 nmol/L). AHAD provides higher sensitivity than other UV or fluorescence derivatizing reagents, such as DNPH^17,18^ and TBA^8,13,15,22^.

### Precision and recovery

Instrumental precision was investigated by analyzing standard MDA solution at three concentrations (1.0, 5.0, and 25 nmol/mL), and five determinations were performed for each concentration. The RSD (%) of peak area were in the range of 0.69–1.06%, which indicated that the instrument has good precision. The analytical precision (intra-day and inter-day) of the method was calculated from a real sample spiked with three concentrations of MDA (1.0, 5.0, and 25 nmol/mL. The intra-day precisions were in the range of 1.36–2.27% and the inter-day precisions were in the range of 2.36–3.92%. The results demonstrated that the method was precise enough for the determination of the MDA in real samples.

The recovery experiments were performed with real samples by spiking three concentrations standard MDA into samples, and three times parallel determination were carried out for each concentration. The preparation and derivatization for the spiked samples were the same as described above. The recoveries were calculated according to the formula of (*x*_1_ − *x*_0_)/*c* × *100%*, in which *x*_*0*_ was the concentration of MDA in the matrix, *x*_1_ was the measured concentration of MDA obtained from the samples spiked with standard MDA and *c* was the known concentration of MDA added to the samples. Good recoveries were obtained in the range of 97–102% with the RSD <4.7% (Table 1).

**Table 1:** Recovery data of MDA in rat prostate.

MDA in sample (μg/g)	Added (μg/g)	Detected (μg/g)	Recovery (%)	RSD (%)
2.52	2.00	4.45	97.2	3.6
2.52	2.50	5.01	99.6	4.6
2.52	3.00	5.55	101.2	4.3

### Advantages of the method

There were several labeling reagents used for the derivatization of MDA in previous reports mentioned above. Compared with the existing method using other derivatization reagents, the developed method with AHAD as pre-column derivatization reagent showed higher sensitivity (Table 2). The LOD in this study is 0.25 nmol/L that is lower than all other methods. Although TAB^8,13,14,22^ is the most commonly used labeling reagent for MDA, it requires high derivatization temperatures (90–100 °C), long derivatization times (60 min). The harsh conditions may cause an artifactual peroxidation of sample constituent. FMOC-hydrazine can react with MDA at a relatively mild reaction temperature (50 °C), but it needs 4 h for the completed reaction^15^. Compared with TBA and FMOC-hydrazine, AHAD has the advantages of relatively mild reaction temperature (80 °C), a fast reaction speed (completed within 30 min). Considering these properties, the HPLC method coupled with AHAD derivatization in this study showed much superiority in simplicity, more sensitivity, relatively mild reaction temperature and shorter derivatization time.

**Table 2:** Critical comparison of the proposed method with reported methods for determination of MDA.

Method	Reagent	Reaction conditions	Linear range (nmol/L)	LOD (nmol/L)	Recovery (%)	Ref
UV-vis spectrophotometry	TBA	100 °C, 60 min	1000–5000	80	66–71	^13^
Fluorescence spectrophotometry	TBA	90 °C, 60 min	14–1400	4.51	95.5–96.8	^8^
HPLC-UV	TBA	100 °C, 15 min	86–9100	34	90–94	^14^
HPLC-UV	DNPH	Room temperature, 30 min	200–20000	200	96.3–99.8	^17^
HPLC-UV	DAN	37 °C, 180 min	76–608	<50	93–108	^19^
HPLC-FLD	FMOC- hydrazine	50 °C, 4 h	500–10000	4	90.2–100.4	^15^
HPLC-FLD	TBA	100 °C, 60 min	150–2430	15	100.4	^22^
HPLC-FLD	AHAD	80 °C, 30 min	2–2500	0.25	95.7–101.2	This work

### Application

The proposed method was applied to analyze the MDA in rat prostate samples. The obtained chromatogram is shown in Fig. 3b. MDA content data from the extracted rat prostate are shown in Table 3. The established method is suitable for the determination of the component from the rat prostate sample with satisfactory results. To the best of our knowledge, this is the first report concerning the HPLC analysis of MDA in the rat prostate. The proposed method can also be used to determinate the MDA in other samples.

**Table 3:** The content of MDA in rat prostate samples.

Sample numbers	MDA (μg/g)
1	2.57 ± 0.14
2	1.97 ± 0.087
3	1.85 ± 0.097
4	2.72 ± 0.12
5	2.54 ± 0.16
6	1.75 ± 0.11

## Conclusions

A fluorescent labeling reagent AHAD was synthesized in this study. AHAD is an excellent derivatization reagent for MDA with acridone as fluorophore and hydrazine as recognition group. A highly sensitive HPLC method for the determination of MDA was developed using AHAD as pre-column derivatization reagent. The optimum derivatization conditions of MDA with the derivatization reagent was at 80 °C for 30 min. The method shows good correlation, precision and accuracy. Compared with the existing methods using other derivatization reagents, the advantages of this method included mild derivatization conditions, simplified sample preparation and higher sensitivity. The proposed method was successfully utilized to determinate MDA in rat prostate. The method also has powerful potential in the determination of MDA in other samples including biological samples, food samples, and so on.

## Supplementary information

Supplementary information.
