# Supplementary material for: Sensitive determination of malondialdehyde in rat prostate by high performance liquid chromatography with fluorescence detection
Source: Sci Rep. 2020 Mar 4;10:3990. doi: 10.1038/s41598-020-61074-3 (PMC7055245; doi:10.1038/s41598-020-61074-3)
Supplement: Supplementary file 1 — Supplementary information. [file 41598_2020_61074_MOESM1_ESM.pdf]

Supplementary information

Title

Sensitive determination of malondialdehyde in rat prostate by high performance liquid chromatography with fluorescence detection

Xiuli Dong · Jiayuan Tang · Xiangming Chen

School of Pharmacy, Binzhou Medical University, Yantai, 264003, China.

Correspondence and requests for materials should be addressed to C.X. (email: xmch913@163.com)

Supplement Fig. 1

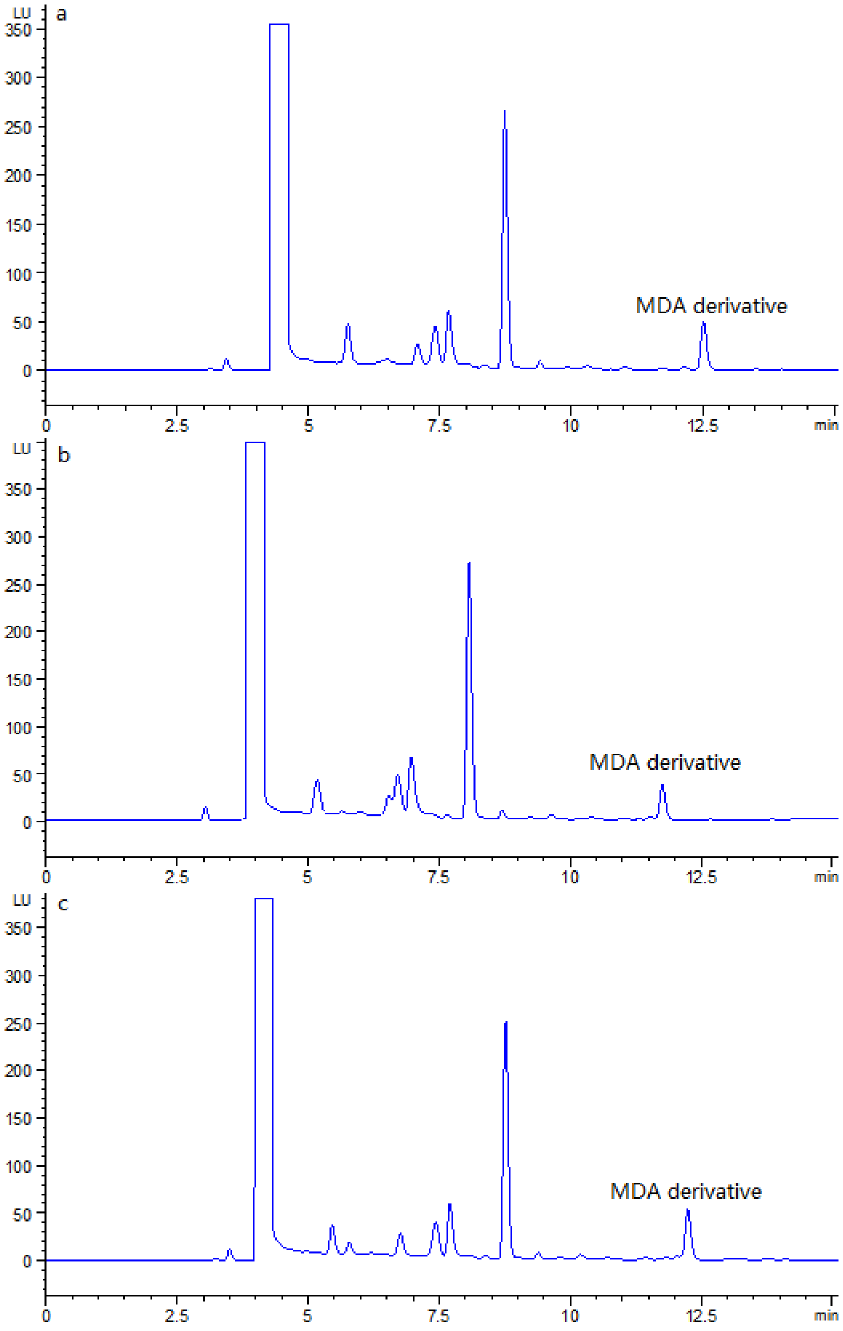

Supplementary Figure caption

Supplement Figure 1. The chromatograms of three columns at the same chromatographic conditions. (a) ZORBAX SB-C18 column; (b) Eclipse XDB C18 column; (c) Eclipse Plus C18 column.
